# Associations Between Polybrominated Diphenyl Ethers Concentrations in Human Placenta and Small for Gestational Age in Southwest China

**DOI:** 10.3389/fpubh.2022.812268

**Published:** 2022-02-08

**Authors:** Yi-jun Liu, Yan Xie, Ying-kuan Tian, Hui Liu, Cai-die He, Song-lin An, Wei Chen, Yuan-zhong Zhou, Xiao-ni Zhong

**Affiliations:** ^1^School of Public Health and Management, Chongqing Medical University, Chongqing, China; ^2^School of Public Health, Zunyi Medical University, Zunyi, China

**Keywords:** polybrominated diphenyl ethers, placenta, pregnancy, small for gestational age, fetal growth

## Abstract

**Background:**

Prenatal exposures to polybrominated diphenyl ethers (PBDEs) may affect fetal growth. Small for gestational age (SGA) is a measure based on birth weight and gestational age at birth and represents a good indicator of fetal growth but it has been used only in a small number of studies. The present study aimed to examine the associations between PBDEs exposure and the risk of SGA among participants from a birth cohort in Southwest China.

**Methods:**

The concentrations of eight common PBDE congeners (BDE-28, BDE47, BDE-99, BDE-100, BDE-153, BDE-154, BDE-183, and BDE-209) in 996 human placental samples collected between May to October 2020 were determined. A questionnaire survey was administered regarding maternal characteristics. The outcome data of the newborns were obtained from the medical record. The Mann–Whitney U test and binomial logistic regression analysis were used to assess associations between PBDEs concentrations (as a continuous or categorical variable) and SGA.

**Results:**

All PBDE congeners were detected in more than 73% of samples. The median concentrations of ΣPBDEs were 10.08 ng/g lipid weight (lw). BDE-209 was the most abundant PBDE congener, contributed 28% to ΣPBDEs. There were 114 (11.4%) SGA infants. The levels of BDE-99, BDE-100, BDE-209, and the total levels of ΣPBDEs in the SGA group were significantly higher than those in the controls. When classifying the PBDEs concentrations as two categories: low and high, high level of ΣPBDEs was associated with increased risk of SGA [odds ratio (OR): 2.203, 95% confidence interval (CI): 1.453–3.340] after adjusting for potential covariates. The association remained significant when stratifying the data by gender of the newborn (OR: 2.572, 95% CI: 1.337–4.947 for boys; OR: 2.385, 95% CI: 1.315–4.325 for girls).

**Conclusion:**

The present study adds to the literature by using placenta to measure PBDEs exposure during pregnancy, and provides evidence that prenatal exposure to PBDEs may be associated with the risk of SGA, at least at the levels of exposure in our population.

## Introduction

Polybrominated diphenyl ethers (PBDEs) are a group of endocrine-disrupting chemicals (EDCs) that have been widely used as brominated flame retardants on electronic equipment, building materials, furniture fillers, foam, textiles materials, and automobile parts (1). Humans can be exposed to PBDEs through diet, inhalation, ingestion, or dermal contact (2). The main source of exposure would be food of animal origin with higher fat content (fish, meat, and dairy products), in which PBDEs tend to accumulate due to their lipophilicity (2). Since PBDEs are used in large quantities and enter human bodies by various pathways, they have been detected in various human samples. In pregnant women, PBDEs transfer across the placenta from maternal circulation and eventually reach the fetus (3, 4). Fetus is more vulnerable and has lower metabolic and immune capabilities to these contaminants than do adults (5, 6). Normal fetal growth is a critical determinant of a healthy pregnancy and influences the long-term health of the offspring. Hence it is important to study the potential effects of PBDEs exposure on fetal growth.

Small for gestational age (SGA) is a measure based on birth weight and gestational age at birth, which is a well-established clinical indicator of intrauterine growth restriction and a strong predictor of short and long-term morbidity (e.g., high risk of suffering from metabolic and cardiovascular diseases in adulthood) and mortality (7), thus allowing for a better estimation of the public health burden of intrauterine growth restriction (8). To our knowledge, only a few epidemiological studies have looked at the association of PBDEs and SGA. Most of them reported a negative correlation between PBDEs levels detected in maternal serum (9), umbilical cord blood (10), colostrum (11), meconium (12) and the risk of SGA. A population-based longitudinal study in New York found no association between PBDEs level in newborn dried blood spots samples and SGA (13). It is still necessary to assess the potential effect of PBDEs on fetus in different population with various human samples.

Some epidemiological studies were also carried out relative to the possible relationship between prenatal exposure to PBDEs and other fetal growth indicators, such as birth weight and gestational age, but the results were discordant (14). For example, Serme-Gbedo et al. evaluated nine epidemiological studies assessing the relationship between PBDEs and birth weight, four of them reported a negative association, while two reported a non-significant negative association, two others reported no statistically significant association, and one reported a negative association for male infants only, and a positive association for female newborns only (15). A recent review concluded that there is not enough evidence to support associations of prenatal exposure to PBDEs with low birth weight and preterm birth (16).

The possible mechanisms underlying the potential effects of PBDEs on fetal growth remains incompletely understood, but could involve impairments in the endocrine system. As a kind of persistent organic pollutants and EDCs, PBDEs can interfere with thyroid system through several pathways (17). Thyroid hormones are critical for development of the fetal and neonatal brain, as well as for many other aspects of pregnancy and fetal growth (18). Another possible mechanism may be alterations in placental development and function by epigenetic dysregulation (DNA methylation, histone modifications and microRNAs) and functional and molecular perturbations to human placental (19–23).

The placenta sits at the interface between the maternal and fetal vascular beds where it mediates nutrient and waste exchange and can act as a protective barrier against toxins and environmental contaminants (24). The fetus is exposed to toxic contaminants only *via* the placenta, which is regarded as a medium that reflects the simultaneous exposure status to persistent organic pollutants of mothers and their fetuses (5, 25). Measurement of PBDEs in human placental tissues is essential to explore their potential fetal exposure.

Therefore, based on data from a birth cohort study in Southwest China, the present study aimed to determine the concentration of PBDEs in human placenta after childbirth, and then assess the associations between PBDEs and the risk of SGA and other fetal grow indicators.

## Materials and Methods

### Study Population, Recruitment, and Data Collection

The study population was selected from the Zunyi Birth Cohort (ZBC), which recruited 1,422 (participate rate: 78%) singleton pregnancy women in the first trimester between May to October 2020. We excluded miscarriages (*n* = 162), stillbirths (*n* = 16), dropped out before delivery (*n* = 138), no records of gestational age, birth weight, or length (*n* = 38), and cases without placenta sample for PBDEs assessment (*n* = 72), leaving 996 (70%) subjects that were entered into the present analysis.

The ZBC study was conducted in four hospitals of Zunyi area from May 2020, aimed at evaluating the effect of environmental factors on pregnancy outcomes and child development. Inclusion criteria for the study included women who were at their first trimester of pregnancy (from gestational week 1 to 12), aged range from 18 to 45 years, with a singleton pregnancy, planning to continue prenatal care and deliver at the hospitals, and intention to live within the study area for the next year. Subjects with a diagnosis of bipolar disorder, schizophrenia, HIV infection, or cancer that resulted in radiation treatment or chemotherapy were excluded from participation. Subjects who fulfill the inclusion and exclusion criteria were asked to sign a written informed consent if they agree to participate.

The cohort women were then followed-up until delivery. The study protocol and informed consent forms were approved by the Ethics Committee of Zunyi Medical University (No. NZKY-007-01).

### Outcome Assessment

The outcome data of the newborns such as birth weight, birth length, gestational age was obtained from medical records. According to the growth reference standard curves of birth weight for newborns of different gestational ages in China (26), SGA refers to an infant born with a birth weight less than the 10th percentile for sex and gestational age-specific birth weight. Infants other than SGA (birth weight greater or equal to the 10th percentile) refer to the control group. Sex and gestational-specific birth weight z-score was calculated by using the same datasets (26).

### Placenta Sample Collection

Placental samples were collected immediately after childbirth by medical staff. In order to obtain a representative sample, four blocks (around 1.5 cm in depth and 1 cm in width) were randomly taken from the maternal and fetal surfaces, respectively, avoiding areas of visible calcification, hematoma, or infarct. The samples were washed in cold phosphate-buffered saline (PBS), dabbed dry, and then kept at −80°C until analysis.

### PBDEs Measurement in Placenta

Eight PBDE congeners (BDE-28, BDE-47, BDE-99, BDE-100, BDE-153, BDE-154, BDE-183, and BDE-209) were selected because they are the most frequently detected congeners in humans (1, 27). The extraction methods used for placenta samples were based on those applied in previous studies (11, 28) with minor modifications. Placenta samples were defrosted, discarded the umbilical cord and all readily removable membranes, and lyophilized. Then the dried placenta was pulverized in a mortar. 2.0 g powder placenta sample was spiked with a known amount of surrogate (50ng BDE-66), and then extracted ultrasonically three times with a hexane/dichloromethane (1:1, vol/vol) mixture. The solvent was removed by centrifugation, and the organic extracts were combined. The lipid content of placenta was then determined gravimetrically by concentrating the extracts to dryness using a nitrogen-blowing instrument (3). Afterward, the lipid content of the placenta sample was removed by a concentrated sulfuric acid and concentrated to 1 ml before a clean-up on a florisil column. The final elute of placenta was concentrated to 50 μL, then 1 μL (2 ng/mL) BDE-77 and 13C-labeled BDE-209 were added into each sample as an internal standard for instrumental analysis.

Prepared placenta samples were analyzed for targeted congeners using gas chromatography-tandem mass spectrometry with electron impact ionization in the multiple reaction monitoring modes (GC-MS/MS; Agilent 8890 gas chromatograph equipped with 7010B tandem mass spectrometer; Agilent Technologies; Santa Clara, CA). 1 μL of the sample was automatically injected in each run through a 15 m DB-5 HT capillary column (0.25 mm i.d., 0.1 mm film thickness). The carrier gas used was high purity helium with a flow rate of 1.2 mL/min, and methane was used as the reaction gas. The oven temperature was programmed as follows: initial temperature of 80°C was held for 1 min; increased to 230°C at a rate of 37°C/min; and then heated up to high temperature of 325°C at a rate of 30°C/min, which was held for 5.3 min.

Some quality control criteria were used to ensure the correct identification and quantification of PBDEs. First, the signal-to-noise ratio must exceed 3:1. Second, the retention times must be within ±5% of those determined from analytical standards. Third, the abundance ratio between quantitative and qualitative ion transitions must be within ±20% of the ratios measured in standards. One procedural blank was tested every ten samples during analysis to make sure that samples were not contaminated. The recovery [mean ± standard deviation (SD)] was 85.8 ± 9.3% for the surrogate standards of BDE-66 in the placentas. The measured lipid content in the placenta samples ranged from 0.7 to 3.5% (mean: 1.6%). The limits of detection (LOD), defined as the mean blank mass plus three SD, were in the range of 0.01–0.27 ng/g of lipid weight (lw) for all congeners except for BDE-209. The LOD for BDE-209 was 0.5 ng/g lw. The detection rate was defined as the percentage of samples with a detectable value, i.e., above LOD.

### Covariates

Information on covariates was derived from questionnaire surveys completed at enrollment and medical records. Several variables were initially considered as confounders based on published literature. Covariates were included in the multivariate models if they were associated with the outcomes and PBDEs at *P* < 0.15 in separate bivariate models. Finally, we included the following confounders: maternal age (continuous), pre-gestational body mass index (BMI, continuous), gestational weight gain (GWG, continuous), parity (primiparous vs. multiparous), maternal educational level (junior middle school or below, senior high school, and college and above), infant sex (male vs. female), and gestational age (continuous).

### Statistical Analysis

We first summarized the background characteristics of mothers and newborns. Pre-pregnancy BMI was calculated by dividing body weight (kg) by height in meters squared. Mean ± SD is provided for continuous statistics and numbers with percentages for categorical variables. For demographic characteristics of the SGA cases and controls, Student's *t*-test was used to identify statistically significant differences in continuous variables, and the χ^2^ test was applied to identify differences in categorical variables.

We then calculated the lipid-adjusted PBDE levels (expressed as ng/g lw) by dividing wet-weight PBDE values by tissue-specific total lipid levels. PBDE concentrations below the LOD were designated as ND (not detectable) and treated as zero for the analysis. The normality of PBDEs concentrations were assessed using the Kolmogorov-Smirnov test and Shapiro-Wilk test. Since the lipid-adjusted PBDEs levels were right-skewed, and logarithmic or natural logarithmic transformation did not improve normality, median, 25th percentile (P_25_), 75th percentile (P_75_) values for PBDE congeners were estimated. The Mann–Whitney *U* test was used to assess the concentrations of PBDEs in placenta between the SGA cases and controls.

To further obtain effect estimates for PBDEs, we modeled PBDEs concentrations as low (below the median concentration) and high (concentrations at or above the median). Logistic regression analysis and multiple linear regression analysis were used to estimate the associations between PBDEs level (low vs. high) and outcomes, after adjustment for important confounders.

We performed sensitivity analysis to evaluate the robustness of our results. First, we reran models after excluding pregnancies complicated by gestational diabetes or hypertension because of their known effects on fetal growth, the model estimates did not change materially (Supplementary Table S1). Therefore, model estimates that include all pregnancies are reported. We also conducted a sensitivity analysis that excluded preterm newborns (<37 gestational weeks) or children born with low birth weight (<2500 g). Last, we explored infant sex differences by examining the association between PBDEs and outcomes stratified by infant sex, as prior researches have reported sex-specific effects of PBDEs (11, 29). All statistical analyses were performed using SPSS version 23.0 (SPSS Inc., Chicago, IL, USA). A two-sided *P* < 0.05 was considered statistically significant.

## Results

### Population Characteristics

Characteristics of the mothers and infants are described in Table 1. Mean maternal age, pre-pregnancy BMI, GWG of the 996 pregnancy women were 27.97 ± 5.24 years, 22.59 ± 3.60 kg/m^2^, 13.63 ± 5.40 kg, respectively. Most women were married or living with a partner (95.8%), and 78.1% were multiparous. Regarding newborns, 49% of the infants were male. The average gestational age, birth weight and length were 39.04 ± 1.39 weeks, 3261.45 ± 439.79 g, 49.98 ± 2.62 cm, respectively. There were 114 (11.4 %) SGA infants. Compared to the controls, significantly lower pre-pregnancy BMI (21.06 ± 2.67 vs. 22.79 ± 3.66 kg/m^2^, *P* < 0.001), and lower GWG (11.88 ± 3.88 vs. 13.85 ± 5.53 kg, *P* < 0.001) were found in the SGA group.

**Table 1:** Participant characteristics.

**Subjects characteristic**	**Total (*n* = 996)**	**SGA (*n* = 114)**	**Control (*n* = 882)**	***P***
Maternal characteristics				
Maternal age (years)	27.97 ± 5.24	27.30 ± 5.06	28.06 ± 5.26	0.146
Pre-pregnancy BMI (kg/m^2^)	22.59 ± 3.60	21.06 ± 2.67	22.79 ± 3.66	<0.001
Gestational weight gain (kg)	13.63 ± 5.40	11.88 ± 3.88	13.85 ± 5.53	<0.001
Han nationality	972 (97.6)	112 (98.2)	860 (97.5)	0.873
Married or living with a partner	954 (95.8)	106 (93.0)	848 (96.1)	0.182
Maternal education level				0.060
Junior middle school and below	546 (54.8)	58 (50.9)	488 (55.3)	
Senior high school	312 (31.3)	32 (28.1)	280 (31.7)	
College and above	138 (13.9)	24 (21.1)	114 (12.9)	
Parity				0.008
Primiparous	218 (21.9)	36 (31.6)	182 (20.6)	
Multiparous	778 (78.1)	78 (68.4)	700 (79.4)	
Family annual income (Yuan)				0.281
<100,000	88 (8.8)	6 (5.3)	82 (9.3)	
100,000–150,000	648 (65.1)	74 (64.9)	574 (65.1)	
≥150,000	260 (26.1)	34 (29.8)	226 (25.6)	
Gestational diabetes mellitus	68 (6.8)	6 (5.3)	62 (7.0)	0.482
Gestational hypertension	56 (5.6)	6 (5.3)	50 (5.7)	0.860
Newborn characteristics				
Male	488 (49.0)	58 (50.9)	430 (48.8)	0.669
Gestational age (weeks)	39.04 ± 1.39	38.63 ± 1.91	39.10 ± 1.29	0.001
Birth weight (g)	3,261.45 ± 439.79	2,660.53 ± 280.13	3,339.12 ± 394.48	<0.001
Birth length (cm)	49.98 ± 2.62	48.40 ± 1.56	50.18 ± 2.65	<0.001

*Data are presented as mean ± standard deviation, or n (%). P-values were calculated in order to compare differences between SGA and the control group*.

*SGA, small for gestational age. BMI, body mass index*.

### PBDEs Levels in Placenta

The concentrations of measured PBDEs (each congener and the sum) in placental tissue are summarized in Table 2. BDE-47 was the most prevalent congener (94.0% detection frequency in placenta sample), while the other PBDE congeners were detected in more than 73% of samples. The sum PBDEs concentrations (ΣPBDEs) for 8 congeners ranged from 1.04 to 248.46 ng/g lw, with a median of 10.08 ng/g lw. BDE-209 was the dominant contributor to the ΣPBDEs, with a median concentration of 2.64 ng/g lw, accounting for 28.0%.

**Table 2:** Concentrations of PBDEs (ng/g lipid wight) in human placenta (*n* = 996).

**Congener**	**Detection rate (%)**	**Contribution (%)**	**Median (P_**25**_, P_**75**_)**	**Range**
BDE-28	73.2	5.9	0.39 (0.04, 1.79)	ND, 39.37
BDE-47	94.0	10.9	0.33 (0.07, 5.50)	ND, 100.70
BDE-99	88.7	9.4	0.52 (0.18, 2.68)	ND, 82.00
BDE-100	90.1	10.3	0.64 (0.20, 3.80)	ND, 57.58
BDE-153	90.9	14.8	1.07 (0.44, 3.55)	ND, 63.63
BDE-154	89.3	9.1	0.54 (0.23, 2.28)	ND, 87.80
BDE-183	91.2	11.7	0.97 (0.35, 2.71)	ND, 77.83
BDE-209	90.1	28.0	2.64 (0.85, 4.36)	ND, 37.30
ΣPBDEs	100.0	100.0	10.08 (5.34, 32.93)	1.04, 248.46

*PBDEs, polybrominated diphenyl ethers*.

### Associations Between Placental PBDE Concentrations and SGA

The median (P_25_, P_75_) concentrations of PBDEs in the SGA group and controls are shown in Table 3. The concentrations of BDE-28, BDE-47, BDE-153, BDE-154, and BDE-183 were not significantly different between the SGA and controls. However, the levels of BDE-99, BDE-100, BDE-209, and the total levels of ΣPBDEs in the SGA group were significantly higher than those in the controls.

**Table 3:** Concentrations of PBDEs (ng/g lipid wight) in SGA and control group.

**Congener**	**SGA (*n* = 114)**	**Control (*n* = 882)**	***P***
BDE-28	0.44 (0.17, 1.40)	0.34 (0.00, 1.98)	0.075
BDE-47	0.60 (0.12,6.25)	0.32 (0.07, 4.88)	0.125
BDE-99	0.99 (0.28, 3.12)	0.49 (0.17, 2.49)	**0.010**
BDE-100	1.04 (0.30, 6.67)	0.57 (0.19, 3.53)	**0.048**
BDE-153	1.30 (0.75, 4.43)	1.06 (0.42, 3.43)	0.062
BDE-154	0.88 (0.29, 2.68)	0.53 (0.23, 2.30)	0.094
BDE-183	1.05 (0.41, 2.88)	0.95 (0.34, 2.68)	0.420
BDE-209	3.29 (1.25, 5.20)	2.58 (0.83, 4.24)	**0.007**
ΣPBDEs	13.6 (7.66, 43.66)	9.64 (5.20, 30.70)	**0.002**

*Data on concentrations are presented as median (25th percentile, 75th percentile)*.

*Significant values P ≤ 0.05 are given in bold*.

*P-values were calculated in order to compare differences between SGA and the control group*.

*PBDEs, polybrominated diphenyl ethers; SGA, small for gestational age*.

When classifying the PBDEs concentrations as two categories: low and high, high level of ΣPBDEs was significantly associated with the risk of SGA [odds ratio (OR): 2.203, 95% confidence interval (CI): 1.453–3.340] after adjusting for potential covariates (Table 4). The association became slightly stronger in male infants than in females (OR: 2.572, 95% CI: 1.337–4.947 for boys; OR: 2.385, 95% CI: 1.315–4.325 for girls), although there was no clear indication for effect modification by sex (p interaction 0.728) (Table 4). When gestational diabetes and hypertension pregnancies, preterm term birth and low birth weight excluded, the significant association of ΣPBDEs with SGA were attenuated but remained significant (Supplementary Table S1).

**Table 4:** Associations between placental PBDEs concentrations (low vs. high) and SGA.

**Congener**	**Girls and boys**		**Boys**		**Girls**		***P* for interaction**
	**Adjusted OR (95% CI)**	***P***	**Adjusted OR (95% CI)**	***P***	**Adjusted OR (95% CI)**	***P***	Sex *PBDEs
BDE-28	1.025 (0.585–1.796)	0.932	0.981 (0.368–2.620)	0.970	0.979 (0.450–2.127)	0.956	0.417
BDE-47	0.807 (0.448–1.456)	0.477	0.839 (0.311–2.264)	0.729	0.768 (0.351–1.678)	0.508	0.727
BDE-99	1.066 (0.588–1.934)	0.833	0.803 (0.309–2.089)	0.653	1.451 (0.651–3.234)	0.363	0.183
BDE-100	1.015 (0.587–1.754)	0.957	1.308 (0.509–3.362)	0.577	0.822 (0.387–1.748)	0.611	0.490
BDE-153	1.049 (0.637–1.727)	0.851	1.338 (0.635–2.818)	0.444	0.737 (0.347–1.565)	0.427	0.273
BDE-154	0.718 (0.420–1.228)	0.226	0.630 (0.273–1.454)	0.279	0.714 (0.338–1.509)	0.378	0.780
BDE-183	0.871 (0.531–1.427)	0.583	0.842 (0.379–1.871)	0.673	0.974 (0.470–2.021)	0.945	0.071
BDE-209	1.236 (0.807–1.892)	0.329	1.509 (0.782–2.911)	0.220	1.106 (0.596–2.052)	0.749	0.613
ΣPBDEs	**2.203 (1.453–3.340)**	**0.001**	**2.572 (1.337–4.947)**	**0.005**	**2.385 (1.315–4.325)**	**0.004**	0.728

*Significant values P ≤ 0.05 are given in bold*.

*Adjusted for maternal age, pre-gestational body mass index, gestational weight gain, parity, educational level, infant sex (as appropriate), and gestational age*.

*PBDEs, polybrominated diphenyl ethers. SGA, small for gestational age. OR, odds ratio; CI, confidence interval*.

### Associations Between Placental PBDE Concentrations and Other Fetal Growth Indicators

Table 5 shows the associations of ΣPBDEs (dichotomous) with birth weight, birth length, gestational age, and birth weight z-score. In adjusted models, high concentration of ΣPBDEs was associated with a 155 g (95% CI: −304, −5) decrease in birth weight, a 0.9 week (95% CI: −1.5, −0.4) reduce in gestational age, and a 0.06 (95% CI: −0.11, −0.02) decrease in birth weight z-score, respectively. No associations were observed between ΣPBDEs and birth length. In sensitivity analysis, exclusion of gestational diabetes and hypertension pregnancies did not change the results (Supplementary Table S1). When we excluded preterm or low birth weight newborn, the associations between ΣPBDEs and gestational age became stronger, while the associations for birth weight, birth length, and birth weight z-score became not statistically significant (Supplementary Table S1).

**Table 5:** Associations between placental ΣPBDEs concentration (low vs. high) and other fetal growth indicators.

**Fetal growth indicators**	**Girls and boys**		**Boys**		**Girls**		***P* for interaction**
	**Adjusted β (95% *CI*)**	***P***	**Adjusted β (95% *CI*)**	***P***	**Adjusted β (95% *CI*)**	***P***	Sex*PBDEs
Birth weight (g)	−154.62 (−303.78, −5.45)	**0.042**	−86.91 (−155.47, −18.35)	**0.013**	−2.39 (−66.63, 61.84)	0.942	0.108
Birth length (cm)	−0.359 (−0.828, 0.11)	0.133	−0.251 (−0.445, −0.057)	**0.011**	−0.114 (−0.335, 0.108)	0.314	0.412
Gestational age (weeks)	−0.943 (−1.475, −0.41)	**0.001**	−0.519 (−0.777, −0.261)	**0.000**	−0.058 (−0.275, 0.158)	0.598	**0.010**
Birth weight z-score	−0.061 (−0.107, −0.016)	**0.009**	−0.039 (−0.061, −0.017)	**0.000**	−0.003 (−0.022, 0.015)	0.721	**0.048**

*Significant values P ≤ 0.05 are given in bold*.

*Adjusted for maternal age, pre-gestational body mass index, gestational weight gain, parity, educational level, infant sex (as appropriate), and gestational age (as appropriate)*.

*CI, confidence interval*.

Significant interaction between PBDEs levels and sex was observed (p-interaction 0.01 and 0.048 for gestational age and birth weight z-score, respectively), thus these analyses were stratified according to infant sex (Table 5). Among boys, higher ΣPBDEs level was associated with a reduction in birth weight (β: −87 g, 95% CI: −155, −18), birth length (β: −0.25 cm, 95% CI: −0.45, −0.06), gestational age (β: −0.52 week, 95% CI: −0.78, −0.26), and birth weight z-score (β: −0.04, 95% CI: −0.06, −0.02). While these associations were not significant among girls.

## Discussion

The present study measured the levels of eight common PBDEs in 996 placenta samples from Southwest China, and examined its associations with the risk of SGA.

Placenta is a stable record of prenatal exposure and has been used previously to evaluate exposure to environmental toxic agents (30). At present, limited studies have measured PBDE concentration in placentas (Supplementary Table S2). The median PBDEs levels in the current study (10.08 ng/g lw) were lower than that found in placenta from the USA (ranging from 19.1 to 33 ng/g lw) (4, 31, 32), and Korea (11.7 ng/g lw) (5), but higher than that in Uganda (7.76 ng/g lw) (33), and European samples (ranging from 1.04 to 2.31 ng/g lw) (3, 25, 34–37). In China, only five studies have reported PBDEs levels in placenta tissue samples (28, 38–41). PBDEs levels in the present study were lower than that found in typical e-waste area [32.25 ng/g lw for Guiyu, Guangdong province (39); 19.5 ng/g lw for Taizhou, Zhejiang province (40)], but higher than that reported from reference areas in China (5.13 ng/g lw for Guangdong province (39); 1.02 ng/g lw for Zhejiang province (40). Caution should be needed in comparing those data, as sampling time points are very crucial for comparative studies of PBDEs due to regulatory actions on PBDEs on domestic and global scales. It is remarkable; however, our study area is not an e-waste recycling area or PBDEs production area in China. Relatively high placental PBDEs levels of our current population indicate that more attention should be paid to the “non-e-waste area.”

The most prominent PBDEs measured in the present study was BDE-209 (contributing 28.0 % to ΣPBDEs). This congener profile was consistent with several studies (3, 5, 28, 33–35, 39), while inconsistent with others in which BDE-47 was a major congener in placenta samples (4, 25, 31, 32, 37, 38, 40, 41). Only one study in Denmark reported BDE-153 (33.6%) as the most abundant congener in human placenta (36). It must be pointed out that the information on BDE-209 is not consistent with studies in China. Some reported higher levels (28, 39), while others did not report or found a lower percentage of detection (38, 40, 41). Analytical difficulties (the fully brominated BDE-209 can degrade into lower brominated congeners), evidence that it is rapidly eliminated from biota, and citation of previous literature focusing on the prevalence of the BDE-47, which may in part explain the discrepancy in the reporting of BDE-209 concentrations in biospecimens (42).

Our results showed an inverse association between PBDEs and SGA risk. The association was significant in the multivariate regression model after adjustment for confounders (Table 4), and was similarly significant when excluded pregnancies with gestational diabetes and hypertension, or preterm and low birth weight newborn from the model (Supplementary Table S1). This support previous findings. In China, a nested case-control study included 101 FGR (defined as the newborn with a birth weight below the 10th percentile for the same gestational age) and 101 healthy newborns that measured PBDEs in maternal serum samples collected during the third trimester and explored the effects of prenatal exposure to PBDEs on birth outcomes. Higher concentrations of BDE-207, −208, −209, and Σ19PBDEs were detected in FGR cases than in controls. After adjusting for confounders, BDE-207 (OR: 1.10, 95% CI: 1.02–1.19) and Σ19PBDEs (OR: 1.01, 95% CI: 1.00–1.02) concentrations in maternal serum were significantly associated with an increased risk of FGR (9). This study also reported an inverse association between PBDEs and placental length, breadth, surface area, indicating that prenatal PBDE exposure may disturb placental growth as the result of reduced placental size (9). Morphological and functional impair of placenta may mediate the adverse effects of PBDEs on fetal growth and development. Another population-based study with 249 mother-newborn pairs also reported that BDE-196-209 (OR: 1.688, 95% CI: 1.024–2.783) and Σ19PBDEs (OR: 2.387, 95% CI: 1.220–4.668) concentrations detected in the umbilical cord blood were associated with increased risk of FGR in newborns (10). The same research team also measured the levels of PBDEs in maternal serum and colostrum in 98 FGR cases (same diagnosis as SGA) and 195 healthy controls. The results indicated that increased concentrations of higher brominated BDEs in maternal serum (OR: 1.010, 95% CI: 1.003–1.018), and low-to moderately brominated BDEs in colostrum (OR: 1.004, 95% CI:1.000–1.009) were associated with increased risk of FGR, which showed an exposure-response relationship (11). In Northwestern Spain, Esther Álvarez-Silvares et al. quantified the concentrations of 50 organic pollutants in meconium from 10 SGA and 40 control newborns, and found that the concentration of BDE-154 in meconium was significantly higher in the SGA group (*P* = 0.049) (12). However, in a New York population-based longitudinal study of 2,065 singleton infants between 2008 and 2010, higher concentrations of BDE-47 in newborn dried blood spots samples were not associated with SGA (OR: 0.99, 95% CI: 0.98–1.00) after adjustment for confounders (13). Differences across studies may be attributed to the different design of the studies, the variation in sample type, geographic location, exposure levels, and methods used for data analysis.

We also observed inverse association between ΣPBDEs concentration with birth weight, gestational age, and birth weight z-score after adjustment for important covariates. These findings align with previous studies (9, 11, 29, 43), but inconsistent with others (15, 44). Interestingly, we found that sex modified the relationship between ΣPBDEs with gestational age and birth weight z-score, with significant reductions in male infants, but null associations in females (P-interaction 0.010 and 0.048 for gestational age and birth weight z-score respectively) (Table 5). Previous studies have reported sex-specific associations between birth outcomes and PBDEs exposure (11, 29). But the underlying mechanisms of these sex-specific differences remains unknown. After further analysis, we found that PBDEs levels were higher in placenta samples from boys than girls (data not shown). Christopher Leonetti et al. reported the same results (45), suggesting infant sex seems to affect the bioaccumulation and metabolism of the placenta during pregnancy. It is plausible that PBDEs have sexually dimorphism biological processes since they are agonists of estrogen receptors and antagonists of androgen receptors (46). Additionally, both the animal and human studies indicate that female adaptations in placental function and growth seem to enable them to be better prepared for adverse events than the male fetuses, male fetus are more sensitive to PBDEs than female (47). However, some studies have also failed to find any significant interaction between sex and PBDEs levels on birth outcome (15, 48–50). We advised caution before concluding that there was actual sex difference between PBDEs exposure and birth outcomes.

Strengths of the current study include a large sample size, and the utilization of placenta to assess PBDEs concentrations, which may reflect the simultaneous exposure to persistent organic pollutants during pregnancy. In addition, this study measured PBDEs exposure during pregnancy in a non-e-waste area in Southwest China. Few studies have assessed PBDEs concentrations in human placenta samples and most of them were conducted in typical e-waste areas. As PBDEs are toxic chemicals that adversely affect human health and the environment around the world, there is a need for more research among understudied populations that investigate how these groups may be disproportionately affected by environmental chemical exposures. One of the potential limitations of our study is that participants were enrolled from a single city and our results may not be generalizable to pregnant women in other locations. In addition, oxidative metabolites of PBDEs, including hydroxylated/methoxylated PBDEs (OH/MeO-PBDEs), which have received extensive attention because of their enhanced toxic effects and effect on fetus (51), were not measured, and should be included in future studies. Finally, while we did adjust for many key covariates, residual confounding may still be a concern.

## Conclusion

The present study adds to the literature by using placenta to measure PBDEs exposure during pregnancy. The PBDEs concentrations detected in placenta samples of our cohort were comparable with other reference areas in China, but lower than those reported in the USA population, and higher than the levels in the European population. BDE-209 was the predominant congener in all detected samples. Our findings provide evidence that prenatal exposure to PBDEs may be associated with the risk of SGA, and reduced gestational age, at least at the levels of exposure in our population.

## Data Availability Statement

The original contributions presented in the study are included in the article/Supplementary Material, further inquiries can be directed to the corresponding author.

## Ethics Statement

The studies involving human participants were reviewed and approved by Ethics Committee of Zunyi Medical University (No. NZKY-007-01). The patients/participants provided their written informed consent to participate in this study.

## Author Contributions

Y-jL and X-nZ: conceptualization. Y-jL, YX, and Y-kT: methodology. Y-jL and HL: formal analysis. C-dH, S-lA, and WC: investigation. Y-zZ and X-nZ: resources and supervision. Y-jL: writing—original draft. Y-jL, YX, Y-zZ, and X-nZ: writing—review & editing. X-nZ: project administration. Y-jL and Y-zZ: funding acquisition. All authors read and approved the final manuscript.

## Funding

This work was supported by the National Key Research and Development Program of China (2018YFC1004302), Science and Technology Program of Guizhou Province (QKH-HBZ [2020]3002), Natural Science Foundation of Guizhou Province (QKH-J[2020]1Y358), and Science and Technology Project of Zunyi (HZ [2019]44). The funders had no role in the design of the study, in the collection, analyses, or interpretation of data, in the writing of the manuscript, or in the decision to publish the results.

## Conflict of Interest

The authors declare that the research was conducted in the absence of any commercial or financial relationships that could be construed as a potential conflict of interest.

## Publisher's Note

All claims expressed in this article are solely those of the authors and do not necessarily represent those of their affiliated organizations, or those of the publisher, the editors and the reviewers. Any product that may be evaluated in this article, or claim that may be made by its manufacturer, is not guaranteed or endorsed by the publisher.
