# Supplementary material for: Associations Between Polybrominated Diphenyl Ethers Concentrations in Human Placenta and Small for Gestational Age in Southwest China
Source: Front Public Health. 2022 Feb 8;10:812268. doi: 10.3389/fpubh.2022.812268 (PMC8863045; doi:10.3389/fpubh.2022.812268)
Supplement: Supplementary file 1 [file Data_Sheet_1.docx]

Supplementary Material

Title：Associations between polybrominated diphenyl ethers concentrations in human placenta and small for gestational age in Southwest China

Yi-jun Liu^1,2^, Yan Xie^2^, Ying-kuan Tian^2^, Hui Liu^1^, Cai-die He^2^, Song-lin An^2^, Wei Chen^2^, Yuan-zhong Zhou^2^, Xiao-ni Zhong^1*^

^1^School of Public Health and Management, Chongqing Medical University, Chongqing 400016, China

^2^School of Public Health, Zunyi Medical University, Zunyi 563000, China

*** Correspondence:**Xiao-ni Zhong
zhongxiaoni@cqmu.edu.cn

Table S 1 Associations between placental ΣPBDEs concentration (low vs. high) with fetal growth indicators

| Fetal growth indicators | All participants, n=996 | | Excluding gestational diabetes and hypertension, n = 888 | | Excluding preterm birth, n = 966 | | Excluding low birth weight, n = 970 | |
| --- | --- | --- | --- | --- | --- | --- | --- | --- |
|  | OR / β (95% *CI*) | *P* | OR / β (95% *CI*) | *P* | OR / β (95% *CI*) | *P* | OR / β (95% *CI*) | *P* |
| SGA ^a^ | 2.141 (1.390-3.296) | **0.001** | 2.232 (1.415, 3.522) | **0.001** | 2.279 (1.466, 3.543) | **0.000** | 1.805 (1.152, 2.829) | **0.010** |
| Birth weight ^b^ (g) | -154.62(-303.78, -5.45) | **0.042** | -41.06 (-87.56, -1.45) | **0.013** | -25.77 (-72.36, 20.82) | 0.278 | -5.03 (-50.20, 40.14) | 0.827 |
| Birth length ^b^ (cm) | -0.359 (-0.828, 0.11) | 0.133 | -0.17 (-0.32, 0.026) | 0.081 | -0.123 (-0.266, 0.021) | 0.093 | -0.032 (-0.164, 0.101) | 0.641 |
| Gestational age ^b^ (weeks) | -0.943 (-1.475, -0.41) | **0.001** | -0.239 (-0.42, -0.058) | **0.010** | -0.226 (-0.366, -0.086) | **0.002** | -0.171 (-0.335, -0.008) | **0.039** |
| Birth weight z-score ^b^ | -0.061 (-0.107, -0.016) | **0.009** | -0.018 (-0.032, -0.003) | **0.016** | -0.009 (-0.022, 0.005) | 0.203 | -0.007 (-0.021, 0.007) | 0.319 |

^a^ Odd ratio (95% confidence interval) for associations between dichotomous PBDEs concentrations and SGA risk.

^b^ β (95% *CI*) associations for associations between dichotomous PBDEs concentrations and outcomes.

Significant values P ≤ 0.05 are given in bold. All models adjusted for maternal age, pre-gestational body mass index, gestational weight gain, parity, educational level, infant sex, and gestational age (as appropriate).

PBDEs, polybrominated diphenyl ethers. SGA, small for gestational age. OR, odds ratio; CI, confidence interval.

**Table S2 PBDE measurements in human placental tissues from various regions**

| **Population location** | | **Year** | **No. of placenta** | **No. of detectable PBDE congeners** | **ΣPBDEs (ng/g lw)** | **Contribution** | **Reference** |
| --- | --- | --- | --- | --- | --- | --- | --- |
| China | Zunyi, Southwest China | 2020 | 996 | 8 | 10.08 (1.04, 248.46) | 209 (28.0 %) | This study |
|  | A typical e-waste area in Guiyu, Guangdong province | 2012 | 69 | 8 | 32.25 (0.89, 516.97)  61.39 ± 85.42 | 209 (40.21%) | (1) |
|  | A reference area in Haojiang, Guangdong province | 2012 | 86 | 8 | 5.13 (0.66, 195.46)  13.03 ± 195.46 | 209 (38.21%) | (1) |
|  | Five major cities in Guangdong province | 2012 | 30 | 15 | 12.7 (4.32, 42.0)  15.8 ± 9.88 | 47 (53.0%) | (2) |
|  | An electronic wastes recycling sites in Zhejiang Province | 2009-2011 | 65 | 9 | 7.0 (3.37, 26.7)  7.90 ± 3.82 | 209 (37.7%) | (3) |
|  | Rural counties in Shanxi Province | 2005-2007 | 130 | 6 | 0.54 (ND, 11.1) | 47 (40.7%) | (4) |
|  | An e-waste site, Taizhou, Zhejiang Province | 2005 | 5 | 36 | 19.5 (1.28, 72.0) | 47 (25.6%) | (5) |
|  | A reference site, 245 km away from Taizhou | 2005 | 5 | 36 | 1.02 (0.59, 1.42) | 47 (24.2%) | (5) |
| Korea | Four cities (Seoul, Anyang, Ansan, and Jeju) | 2011 | 108 | 6 | 11.7 (1.21, 427)  22.0 ± 53.1 | 209 (88%) | (6) |
| USA | Northern California and the Central Valley | 2014-2016 | 180 | 5 | Median: 26 | 47 (.36.5%) | (7) |
|  | Durham County, North Carolina | 2010-2011 | 102 | 6 | 19.1 (0.54, 528) | 47 (26.4%) | (8) |
|  | New York, Wisconsin, California* | 2010-2012 | 42 | 10 | 33 (4.26, 172.3)  44.6 ± 40.8 | 47 (37%) | (9) |
| Uganda | Kampala | 2018 | 30 | 24 | 7.76 (0.25, 30.9) | 209 (40.5%) | (10) |
| Spain | Ourense | 2016 | 88 | 7 | 8.6 ± 12 | 47 (23%) | (11) |
|  | Asturias | 2004-2007 | 49 | 6 | Median: 2.3 | 209 | (12) |
|  | Madrid | 2003–2004 | 30 | 15 | 1.9 (0.19, 9.7) | 209 (52.6%) | (13) |
| Denmark | Copenhagen University Hospital | 2007 | 50 | 12 | 2.31 (0.51, 17.10) | 209 (49.4%) | (14) |
|  | Copenhagen | 1997-2001 | 129 | 5 | Median:1.28 | 153 (33.6%) | (15) |
| Finland | Kuopio University Hospital | 2004-2005 | 130 | 9 | Median: 1.04 | 47 (37.5%) | (16) |
|  | Turku | 1997-2001 | 56 | 5 | Median: 1.16 | 47 (50.8%) | (15) |

Data are presented as median (min, max) and mean ± standard deviation unless stated otherwise. ND, the detection frequency was too low to allow statistical treatment or no detect.

*The concentration value in this paper was presented as pg/g wet weight. We calculated lw (ng/g) = ww (pg/g)/1000/lipid%, where lipid% is the median or average lipid content, a value of 1% is used as the lipid% is not reported in the literature paper.

**References**

1. Xu L, Huo X, Zhang Y, Li W, Zhang J, Xu X. Polybrominated diphenyl ethers in human placenta associated with neonatal physiological development at a typical e-waste recycling area in China. *Environmental pollution (Barking, Essex : 1987)* (2015) 196:414-22. Epub 2014/12/04. doi: 10.1016/j.envpol.2014.11.002.

2. Chen ZJ, Liu HY, Cheng Z, Man YB, Zhang KS, Wei W, et al. Polybrominated diphenyl ethers (PBDEs) in human samples of mother-newborn pairs in South China and their placental transfer characteristics. *Environment international* (2014) 73:77-84. Epub 2014/08/05. doi: 10.1016/j.envint.2014.07.002.

3. Zhao Y, Ruan X, Li Y, Yan M, Qin Z. Polybrominated diphenyl ethers (PBDEs) in aborted human fetuses and placental transfer during the first trimester of pregnancy. *Environmental science & technology* (2013) 47(11):5939-46. Epub 2013/04/30. doi: 10.1021/es305349x.

4. Ma J, Qiu X, Ren A, Jin L, Zhu T. Using placenta to evaluate the polychlorinated biphenyls (PCBs) and polybrominated diphenyl ethers (PBDEs) exposure of fetus in a region with high prevalence of neural tube defects. *Ecotoxicology and environmental safety* (2012) 86:141-6. Epub 2012/10/02. doi: 10.1016/j.ecoenv.2012.09.005.

5. Leung AO, Chan JK, Xing GH, Xu Y, Wu SC, Wong CK, et al. Body burdens of polybrominated diphenyl ethers in childbearing-aged women at an intensive electronic-waste recycling site in China. *Environmental science and pollution research international* (2010) 17(7):1300-13. Epub 2010/03/20. doi: 10.1007/s11356-010-0310-6.

6. Jeong Y, Lee S, Kim S, Park J, Kim HJ, Choi G, et al. Placental transfer of persistent organic pollutants and feasibility using the placenta as a non-invasive biomonitoring matrix. *The Science of the total environment* (2018) 612:1498-505. Epub 2017/09/16. doi: 10.1016/j.scitotenv.2017.07.054.

7. Varshavsky JR, Sen S, Robinson JF, Smith SC, Frankenfield J, Wang Y, et al. Racial/ethnic and geographic differences in polybrominated diphenyl ether (PBDE) levels across maternal, placental, and fetal tissues during mid-gestation. *Scientific reports* (2020) 10(1):12247. Epub 2020/07/24. doi: 10.1038/s41598-020-69067-y.

8. Leonetti C, Butt CM, Hoffman K, Miranda ML, Stapleton HM. Concentrations of polybrominated diphenyl ethers (PBDEs) and 2,4,6-tribromophenol in human placental tissues. *Environment international* (2016) 88:23-9. Epub 2015/12/25. doi: 10.1016/j.envint.2015.12.002. PubMed PMID: 26700418.

9. Nanes JA, Xia Y, Dassanayake R, Jones RM, Li A, Stodgell CJ, et al. Selected persistent organic pollutants in human placental tissue from the United States. *Chemosphere* (2014) 106:20-7. Epub 2014/02/04. doi: 10.1016/j.chemosphere.2013.12.080.

10. Matovu H, Ssebugere P, Sillanpää M. Prenatal exposure levels of polybrominated diphenyl ethers in mother-infant pairs and their transplacental transfer characteristics in Uganda (East Africa). *Environmental pollution (Barking, Essex : 1987)* (2020) 258:113723. Epub 2019/12/24. doi: 10.1016/j.envpol.2019.113723.

11. Fernández-Cruz T, Álvarez-Silvares E, Domínguez-Vigo P, Simal-Gándara J, Martínez-Carballo E. Prenatal exposure to organic pollutants in northwestern Spain using non-invasive matrices (placenta and meconium). *The Science of the total environment* (2020) 731:138341. Epub 2020/05/15. doi: 10.1016/j.scitotenv.2020.138341.

12. Vizcaino E, Grimalt JO, Fernández-Somoano A, Tardon A. Transport of persistent organic pollutants across the human placenta. *Environment international* (2014) 65:107-15. Epub 2014/02/04. doi: 10.1016/j.envint.2014.01.004.

13. Gómara B, Herrero L, Ramos JJ, Mateo JR, Fernández MA, García JF, et al. Distribution of polybrominated diphenyl ethers in human umbilical cord serum, paternal serum, maternal serum, placentas, and breast milk from Madrid population, Spain. *Environmental science & technology* (2007) 41(20):6961-8. Epub 2007/11/13. doi: 10.1021/es0714484.

14. Frederiksen M, Thomsen M, Vorkamp K, Knudsen LE. Patterns and concentration levels of polybrominated diphenyl ethers (PBDEs) in placental tissue of women in Denmark. *Chemosphere* (2009) 76(11):1464-9. Epub 2009/08/18. doi: 10.1016/j.chemosphere.2009.07.017.

15. Main KM, Kiviranta H, Virtanen HE, Sundqvist E, Tuomisto JT, Tuomisto J, et al. Flame retardants in placenta and breast milk and cryptorchidism in newborn boys. *Environmental health perspectives* (2007) 115(10):1519-26. Epub 2007/10/17. doi: 10.1289/ehp.9924.

16. Leino O, Kiviranta H, Karjalainen AK, Kronberg-Kippilä C, Sinkko H, Larsen EH, et al. Pollutant concentrations in placenta. *Food and chemical toxicology : an international journal published for the British Industrial Biological Research Association* (2013) 54:59-69. Epub 2011/11/08. doi: 10.1016/j.fct.2011.10.058.
